# CYP24A1 in Small Intestinal Vitamin D Metabolism and Clinical Implications

**DOI:** 10.3390/nu17213348

**Published:** 2025-10-24

**Authors:** Agnieszka Nowacka, Maciej Śniegocki, Dominika Bożiłow, Ewa A. Ziółkowska

**Affiliations:** 1 Department of Neurosurgery, Nicolaus Copernicus University in Toruń, Collegium Medicum in Bydgoszcz, ul. Curie Skłodowskiej 9, 85-094 Bydgoszcz, Poland; sniegocki@cm.umk.pl; 2 Anaesthesiology and Intensive Care Clinical Ward, The 10th Military Research Hospital and Polyclinic, ul. Powstańców Warszawy 5, 85-681 Bydgoszcz, Poland; bozilow@wp.pl; 3 Department of Pediatrics, School of Medicine, Washington University in St. Louis, St. Louis, MO 63110, USA

**Keywords:** CYP24A1, vitamin D, vitamin D metabolism, enterocytes, cytochrome P450 enzyme, P450, VDR signaling, gut inflammation, calcium absorption, calcium

## Abstract

CYP24A1, a mitochondrial cytochrome P450 enzyme, plays a critical role in the catabolism of active vitamin D metabolites and is a key regulator of local vitamin D signaling in the small intestine. While traditionally studied in the context of renal physiology, increasing evidence highlights its distinct regulatory mechanisms and functional significance within the intestinal epithelium. This review explores the molecular architecture, tissue-specific expression patterns, and multifactorial regulation of CYP24A1 in enterocytes, encompassing nuclear receptor signaling, epigenetic and post-transcriptional control, and environmental influences such as inflammation, diet, and the gut microbiota. We discuss how intestinal CYP24A1 modulates the expression of vitamin D target genes involved in transcellular calcium absorption and epithelial barrier function, and how its dysregulation contributes to gastrointestinal disorders including inflammatory bowel diseases, celiac disease, microbiota dysbiosis, and colorectal cancer. In addition, we examine preclinical and translational evidence supporting CYP24A1 as a potential therapeutic target. Emerging strategies such as selective enzyme inhibitors, microbiota modulation, RNA-based technologies, and personalized supplementation approaches are considered in the context of restoring local vitamin D bioactivity and mineral homeostasis. Together, this review underscores the clinical importance of intestinal CYP24A1 and highlights novel opportunities for targeted interventions in vitamin D-responsive gastrointestinal pathologies.

## 1. Introduction

Vitamin D is widely recognized for its role in regulating calcium and phosphate homeostasis. However, accumulating evidence highlights its broader physiological functions, including modulation of the immune system, maintenance of epithelial integrity, and regulation of cellular proliferation and differentiation [1,2,3,4]. These effects are primarily mediated by the active metabolite 1,25-dihydroxyvitamin D_3_ [1,25(OH)_2_D_3_] through its interaction with the nuclear vitamin D receptor (VDR). The mitochondrial enzyme CYP24A1 plays a key role in controlling vitamin D activity by catabolizing 1,25(OH)_2_D_3_ and related metabolites, thereby ensuring hormonal balance and preventing toxic accumulation [5,6,7,8,9,10].

While the role of CYP24A1 in systemic vitamin D metabolism, particularly within renal tissues, has been well studied, its function in the small intestine remains incompletely characterized (Table 1). Enterocytes express CYP24A1 and other components necessary for local vitamin D metabolism, suggesting that the gut epithelium is capable of autonomous regulation. This may be essential for responding to dietary calcium fluctuations, regulating epithelial renewal, and modulating local immune responses [2,10,11]. Despite these potential roles, the regulatory mechanisms and clinical significance of intestinal CYP24A1 have received limited attention in the literature.

Recent studies have shown that CYP24A1 expression in the intestine is regulated by a complex network of systemic hormones, local inflammatory signals, microbiota-derived metabolites, and epigenetic modifications [9,10,12]. Dysregulation of this enzyme has been observed in several gastrointestinal disorders, including inflammatory bowel disease, celiac disease, short bowel syndrome, and colorectal cancer [4,10,13,14]. These findings raise the possibility that CYP24A1 may contribute to both the pathophysiology and treatment responsiveness of these conditions.

This review aims to provide an up-to-date synthesis of the literature on CYP24A1 in the small intestine. We discuss its structural and regulatory features, summarize mechanisms of transcriptional and post-transcriptional control, and explore its expression across different intestinal segments. We also examine how CYP24A1 is altered in gastrointestinal diseases and consider its potential as a therapeutic target. By consolidating current knowledge, this article seeks to clarify the physiological relevance of intestinal CYP24A1 and identify areas where further research is needed.

## 2. CYP24A1—Structure, Function, and Regulation

### 2.1. Structural and Biochemical Features of CYP24A1

**Figure 1:**
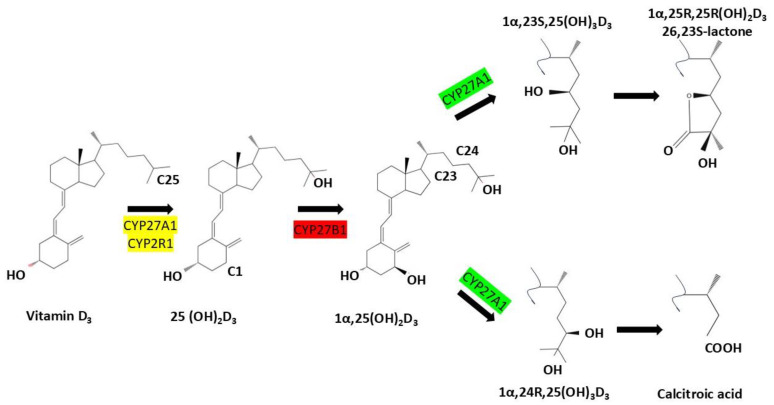
Metabolic activation and degradation pathways of vitamin D_3_: Vitamin D_3_ (cholecalciferol) undergoes sequential hydroxylation steps catalyzed by cytochrome P450 enzymes. CYP27A1 and CYP2R1 convert it to 25-hydroxyvitamin D_3_ [25(OH)D_3_], followed by CYP27B1-mediated activation to 1α,25-dihydroxyvitamin D_3_ [1α,25(OH)_2_D_3_]. CYP24A1 then initiates degradation via C23- or C24-hydroxylation, forming metabolites such as 1,24,25(OH)_3_D_3_, lactone derivatives, and ultimately calcitroic acid. These catabolic routes regulate tissue-level vitamin D activity and prevent toxicity.

CYP24A1 is a mitochondrial cytochrome P450 enzyme that plays a central role in regulating vitamin D homeostasis through catabolism of active vitamin D metabolites. Structurally, CYP24A1 possesses a canonical P450 fold with a heme prosthetic group necessary for monooxygenase activity, as shown by crystallographic studies at 2.5 Å resolution [2,10,12,15,16,17]. Synthesized in the cytosol as a ~60 kDa precursor, the protein is targeted to mitochondria via an *N*-terminal sequence and the TOM20 import complex, where it is processed into a mature ~55 kDa form embedded in the inner mitochondrial membrane. This localization is essential for its function in tissues such as the kidney and small intestine. CYP24A1 catalyzes sequential hydroxylation of 1,25-dihydroxyvitamin D_3_ [1,25(OH)_2_D_3_] and 25-hydroxyvitamin D [25(OH)D] through the C24-oxidation pathway, generating inactive metabolites like 1,24,25-trihydroxyvitamin D_3_ and calcitroic acid, which are excreted via bile or urine. This enzymatic activity serves as a protective feedback mechanism that prevents excessive vitamin D signaling and hypercalcemia [10,12,15,17,18,19] (Figure 1).

### 2.2. Mechanisms Regulating CYP24A1 Expression

Expression of CYP24A1 is tightly regulated by intracellular levels of 1,25(OH)_2_D_3_ via activation of the vitamin D receptor (VDR), which heterodimerizes with retinoid X receptor (RXR) to bind vitamin D response elements (VDREs) within the CYP24A1 promoter [10,17,20]. This ligand-dependent transcriptional control is further modulated by hormones such as parathyroid hormone (PTH), which suppresses CYP24A1, and fibroblast growth factor 23 (FGF23), which enhances its expression in the kidney to limit vitamin D excess and promote phosphate excretion [8,19,21,22,23,24]. These endocrine feedback loops form part of a broader bone–kidney–parathyroid axis that becomes dysregulated in disorders like chronic kidney disease (CKD). Additionally, epigenetic mechanisms such as DNA methylation and histone modifications (e.g., H3K4me2, H3K9ac) modulate CYP24A1 transcriptional activity, particularly in cancer and inflamed tissues. Promoter hypermethylation can silence CYP24A1 expression, while pharmacological inhibition of DNA methyltransferases or histone deacetylases has been shown to restore transcriptional responsiveness to calcitriol [17,19,25,26,27].

### 2.3. Post-Transcriptional and Tissue-Specific Regulation

Post-transcriptionally, several microRNAs—including miR-27, miR-30b-5p, and miR-224—have been identified as negative regulators of CYP24A1 mRNA, further contributing to fine-tuning protein levels in response to physiological or pathological stimuli. Moreover, inflammatory signals such as lipopolysaccharide (LPS) can suppress CYP24A1 while transiently upregulating CYP27B1, enhancing local 1,25(OH)_2_D_3_ synthesis during immune responses. These mechanisms collectively integrate endocrine, immune, and environmental signals to ensure context-specific regulation of vitamin D metabolism [12,17,28]. Structural models, including those based on the rat Cyp24a1 homolog, have provided insight into the impact of mutations and exon deletions on enzymatic function, with several pathogenic variants linked to disorders like idiopathic infantile hypercalcemia. Recent data also suggest the possible existence of alternative CYP24A1 isoforms generated through alternative splicing or post-translational modification, though their functional relevance remains under investigation [9,29,30,31,32]. These isoforms, if functionally active, could potentially exhibit altered enzymatic activity, different substrate specificity toward vitamin D metabolites, or even act as dominant-negative regulators that compete with wild-type CYP24A1 for binding partners or subcellular localization. Such variants could also display tissue-specific expression patterns that fine-tune local vitamin D catabolism in response to developmental or pathological cues.

Tissue-specific expression patterns further demonstrate that while the kidney maintains high basal CYP24A1 levels for systemic control, extra-renal sites like the small intestine display more dynamic regulation in response to dietary, developmental, and inflammatory cues. For example, intestinal CYP24A1 is inducible by VDR signaling and appears to influence both local calcium absorption and epithelial barrier function. Mouse models with tissue-specific Cyp24a1 deletion confirm its local physiological relevance. Together, these findings underscore CYP24A1’s pivotal role as a metabolic gatekeeper and transcriptional integrator, essential for maintaining vitamin D balance and mineral homeostasis across diverse physiological contexts [9,12,17,28,29].

## 3. Expression and Regulation of CYP24A1 in the Small Intestine

### Spatial Expression Along the Intestinal Tract

Within the small intestine, CYP24A1 expression is dynamically regulated along the duodenum, jejunum, and ileum, reflecting distinct regional roles in vitamin D metabolism and calcium absorption. The duodenum, the principal site of transcellular calcium uptake, exhibits exceptional responsiveness to 1,25-dihydroxyvitamin D_3_ [1,25(OH)_2_D_3_], with Cyp24a1 mRNA levels increasing over 370,000-fold after hormone administration—far exceeding renal induction [33,34,35]. Despite low basal levels, CYP24A1 is rapidly upregulated across villus and crypt cells, forming a potent local feedback loop to degrade excess 1,25(OH)_2_D_3_ and maintain epithelial homeostasis. This process, largely driven by vitamin D receptor (VDR) activation, is also modulated by systemic hormones like parathyroid hormone (PTH) and fibroblast growth factor 23 (FGF23) [10,12,36].

In the jejunum, CYP24A1 is similarly inducible and plays a crucial role in regulating TRPV6 and calbindin-D expression to fine-tune calcium absorption in response to dietary intake. Local differences in hormonal sensitivity may allow this segment to adapt independently of renal control. Mutations or epigenetic suppression of CYP24A1 in this region have been linked to hyperabsorption and nephrolithiasis [10,12,37,38].

**Figure 2:**
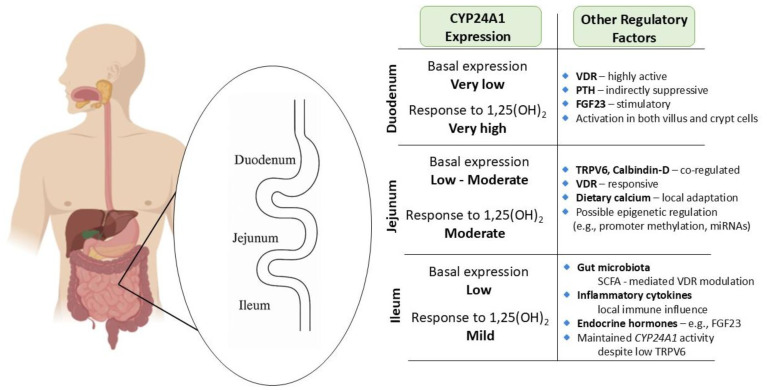
Segment-specific expression and regulation of CYP24A1 in the small intestine: Illustration of CYP24A1 expression levels and regulatory factors across different segments of the small intestine. Basal expression is lowest in the duodenum but highly inducible by 1,25-dihydroxyvitamin D_3_ [1,25(OH)_2_D_3_]. The jejunum exhibits moderate basal and inducible expression, with strong responsiveness to dietary calcium and VDR signaling. The ileum demonstrates mild responsiveness and is primarily regulated by microbial and inflammatory cues. Segment-specific differences reflect the regional specialization in calcium absorption and local vitamin D metabolism, and highlight the complex interplay of endocrine, epigenetic, and environmental modulators.

Although less prominent in the ileum, CYP24A1 remains functionally relevant, helping to regulate vitamin D levels where TRPV6 expression is reduced. Its activity in ileal epithelial cells is shaped by endocrine, microbial, and inflammatory cues, positioning this segment as a site of metabolic fine-tuning. Altogether, the segment-specific regulation of CYP24A1 enables the intestine to adaptively control vitamin D catabolism and maintain mineral and mucosal homeostasis [39,40] (Figure 2).

## 4. Mechanisms Regulating Intestinal CYP24A1

### 4.1. Nuclear Receptor-Mediated Control

Nuclear receptors are central to the regulation of CYP24A1 expression in the small intestine, acting as integrators of hormonal and metabolic cues to fine-tune local vitamin D catabolism. The vitamin D receptor (VDR), upon activation by 1,25-dihydroxyvitamin D_3_ [1,25(OH)_2_D_3_], heterodimerizes with the retinoid X receptor (RXR) and binds to vitamin D response elements (VDREs) within the CYP24A1 promoter. This interaction initiates transcription of CYP24A1, promoting the degradation of active vitamin D metabolites to maintain calcium–phosphate homeostasis and prevent hormonal excess [9,10,12,19,20,41]. While the kidney remains the principal site of systemic vitamin D clearance, intestinal epithelial cells also require tightly regulated, localized mechanisms—especially during dietary fluctuations. These cells exhibit high sensitivity to 1,25(OH)_2_D_3_ and rapidly adjust CYP24A1 expression accordingly. Beyond VDR, other nuclear receptors contribute to this regulatory network. For instance, glucocorticoid receptors, when activated by agents such as dexamethasone, have been shown to upregulate CYP24A1 expression in both renal and intestinal tissues, illustrating cross-talk between corticosteroid and vitamin D pathways [10,17,18,20,26,27]. Moreover, receptors activated by microbial metabolites and dietary components may further modulate CYP24A1 in a context-dependent manner, though these mechanisms are less well-defined. The chromatin landscape—comprising accessible promoters and distal enhancers—also plays a key role in shaping this response, enabling rapid transcriptional activation. However, during inflammation, VDR expression is markedly suppressed, impairing CYP24A1 induction and contributing to disordered vitamin D metabolism. This disruption compromises the integrity of the epithelial barrier and increases susceptibility to gastrointestinal disorders, further highlighting the importance of nuclear receptor-mediated regulation in maintaining gut homeostasis [2,13,14,40,42,43,44,45,46].

### 4.2. Epigenetic and microRNA-Mediated Regulation

Epigenetic mechanisms provide an additional layer of control over CYP24A1 expression in enterocytes, enabling dynamic responses to environmental, inflammatory, and microbial cues. One of the most prominent forms of epigenetic regulation is DNA methylation. In colorectal cancer, for example, hypermethylation of the CYP24A1 promoter correlates with suppressed gene and protein expression. This epigenetic silencing may shift the balance toward elevated 1,25(OH)_2_D_3_ bioavailability, thereby influencing downstream vitamin D target genes involved in calcium and phosphate transport [17,26,27,29]. Beyond methylation, histone modifications and non-coding RNAs—particularly microRNAs such as miR-27, miR-30b-5p, and miR-224—contribute to post-transcriptional regulation. These microRNAs may act directly by binding CYP24A1 mRNA or indirectly by modulating VDR and its cofactors. Inflammatory signals, including TNF-α, can stimulate the expression of such regulatory RNAs, linking immune activation to altered vitamin D metabolism [17,27,47,48,49,50,51]. The gut microbiota further influences this epigenetic landscape. Microbial metabolites such as short-chain fatty acids (SCFAs) can modify histone acetylation in epithelial cells, impacting CYP24A1 transcriptional activity. These interactions illustrate a complex and adaptive framework whereby environmental and microbial factors shape local vitamin D responsiveness. Importantly, pharmacologic targeting of these epigenetic regulators—using demethylating agents or histone deacetylase inhibitors—may restore physiologic CYP24A1 expression and vitamin D sensitivity, especially in inflammatory or neoplastic conditions of the intestine [12,14,17,38,39,52,53].

### 4.3. The Microbiota-Inflammation Axis in CYP24A1 Regulation

Microbial-derived metabolites and inflammatory signaling form a tightly connected axis that critically modulates intestinal CYP24A1 expression. Under healthy conditions, microbial communities support intestinal homeostasis through the production of metabolites such as SCFAs, which contribute to epigenetic regulation of vitamin D pathway genes [13,14,54,55]. However, in diseases like inflammatory bowel disease (IBD), dysbiosis leads to elevated lipopolysaccharide (LPS) levels and increased cytokine production, disrupting the expression of key vitamin D hydroxylases, including CYP27B1 and CYP24A1. Inflammation not only reduces substrate availability by suppressing CYP27B1 but also downregulates VDR expression, compromising feedback regulation of CYP24A1 [2,10,16,51]. This suppression is exacerbated by diminished production of VDR-induced antimicrobial peptides, particularly in Crohn’s disease. Additionally, environmental factors such as calcium intake, micronutrient status, and hydration may influence microbial activity and, consequently, CYP24A1 expression [17,56]. Mouse models further illustrate the significance of this axis: VDR-deficient mice demonstrate heightened susceptibility to colitis and increased expression of inflammatory mediators like IFN-γ and IL-17, along with dysregulated CYP24A1. Some pathogenic bacteria can even bind or degrade VDR, directly interfering with its transcriptional activity. The resulting loss of regulatory balance forms a vicious cycle of inflammation, VDR suppression, and CYP24A1 upregulation. These insights suggest that therapeutic strategies aimed at modulating the microbiota or enhancing VDR activity could help normalize CYP24A1 expression and restore intestinal equilibrium [5,12,13,57,58,59,60].

Chronic inflammation in the gut has profound effects on vitamin D metabolism, primarily through its impact on CYP24A1 expression. Inflammatory cytokines such as IL-6 and TNF-α, commonly elevated in IBD, interfere with normal hydroxylase function, including that of CYP2R1, CYP27B1, and CYP24A1. These inflammatory mediators decrease circulating and local 25(OH)D levels and promote the degradation of 1,25(OH)_2_D_3_, resulting in impaired calcium–phosphate absorption and compromised epithelial function [4,17,18]. Vitamin D normally acts via the VDR to dampen inflammatory signaling and preserve barrier integrity. However, during inflammation, reduced VDR expression weakens this protective mechanism and enhances CYP24A1 activity, further lowering bioactive vitamin D levels. In animal models, this results in a feed-forward loop where immune activation leads to elevated pro-inflammatory cytokines, which in turn drive further VDR suppression and CYP24A1 dysregulation [17,18,50,61]. Additional disruption occurs through interference with autophagy-related genes like ATG16L1, which are essential for epithelial repair and are themselves regulated by both cytokines and vitamin D signaling. Genetic polymorphisms in VDR and immune regulatory genes further influence individual susceptibility to inflammation-induced CYP24A1 imbalances. Clinically, low serum vitamin D levels correlate with increased IBD severity, emphasizing the functional importance of this regulatory axis [43,62,63].

### 4.4. Hormonal Regulation in the Gut

**Figure 3:**
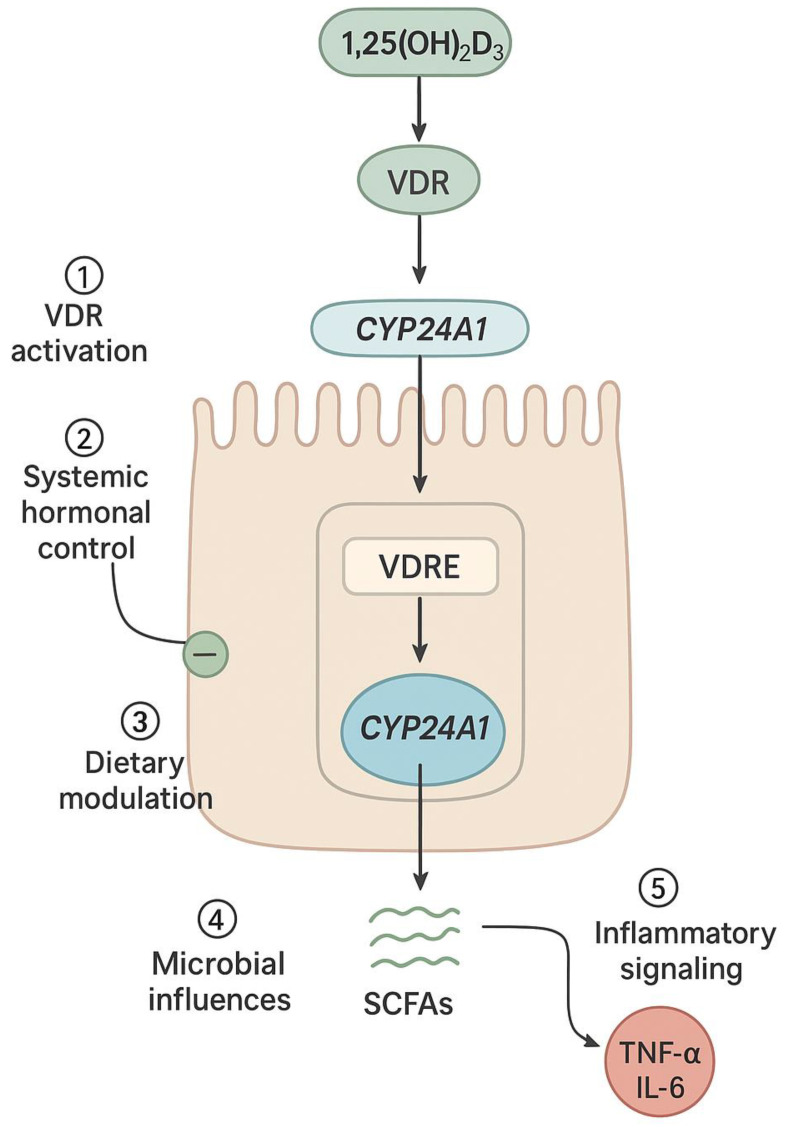
Intestine-specific hormonal and environmental regulation of CYP24A1 in enterocytes: Schematic representation of an intestinal epithelial cell showing the convergence of multiple regulatory inputs on CYP24A1 expression. These include: (1) VDR activation by 1,25(OH)_2_D_3_ leading to transcriptional upregulation via VDREs in the CYP24A1 promoter; (2) systemic hormonal signals including PTH (suppressive) and FGF23 (stimulatory); (3) dietary factors such as calcium and phosphate intake; (4) microbial metabolites including short-chain fatty acids (SCFAs) that modulate epigenetic marks; and (5) inflammatory cytokines (TNF-α, IL-6) that disrupt VDR signaling. CYP24A1 then catalyzes the degradation of 1,25(OH)_2_D_3_ to calcitroic acid, forming a local feedback loop that maintains intestinal vitamin D homeostasis.

Systemic hormones play a vital role in modulating intestinal CYP24A1 expression, complementing local and environmental regulation. The interaction between parathyroid hormone (PTH), fibroblast growth factor 23 (FGF23), and 1,25(OH)_2_D_3_ represents a tightly coordinated endocrine axis that governs vitamin D metabolism in both the kidney and intestine. In the renal system, PTH enhances CYP27B1 and inhibits CYP24A1 to boost active vitamin D production, while FGF23 and 1,25(OH)_2_D_3_ stimulate CYP24A1 expression to reduce circulating levels in a feedback loop [9,17,64]. Although intestinal CYP24A1 regulation is less well-characterized, enterocytes express VDR and similar transcriptional machinery to renal cells. Studies suggest that 1,25(OH)_2_D_3_ induces intestinal CYP24A1 via autocrine or paracrine signaling, helping to fine-tune vitamin D action locally. This mechanism appears highly responsive to dietary calcium levels. In calcium deficiency, CYP24A1 expression is downregulated to conserve 1,25(OH)_2_D_3_, promoting increased calcium absorption. In contrast, excess calcium intake upregulates CYP24A1 to accelerate hormone degradation and prevent hypercalcemia [27,35,65]. Dietary phosphate exerts a similar influence through FGF23-dependent pathways. This hormonal responsiveness underscores the intestine’s active role in maintaining mineral balance. Promoter and enhancer regions responsive to PTH, FGF23, and 1,25(OH)_2_D_3_ allow for tissue-specific fine-tuning of CYP24A1 expression. Additionally, local factors such as microbial products and inflammatory mediators are likely integrated into this hormonal framework, further shaping enterocyte behavior under both physiological and pathological conditions [10,27,46,65] (Figure 3).

### 4.5. Dietary and Environmental Factors

Environmental and dietary inputs constitute essential modulators of CYP24A1 activity in the intestine. The intestinal epithelium, rich in VDR, responds dynamically to variations in dietary vitamin D, calcium, phosphate, and sunlight exposure [11,35,66,67]. These factors influence systemic 1,25(OH)_2_D_3_ levels and, consequently, VDR activation, which promotes CYP24A1 transcription as part of a homeostatic feedback mechanism. Among dietary factors, calcium intake plays a particularly prominent role. Under conditions of calcium scarcity, CYP24A1 expression is suppressed to preserve 1,25(OH)_2_D_3_ and enhance intestinal absorption. Conversely, elevated calcium intake stimulates CYP24A1 activity, promoting degradation of the hormone to avoid excessive mineral uptake. Phosphate intake similarly influences CYP24A1 through FGF23 signaling pathways. Sunlight exposure, particularly UVB, increases endogenous vitamin D synthesis and indirectly promotes intestinal CYP24A1 expression [12,68,69]. Additional environmental elements—including hydration status, exposure to pollutants, and availability of micronutrients—can influence vitamin D metabolism by altering endocrine or epigenetic regulation. Furthermore, chronic conditions such as chronic kidney disease (CKD), often influenced by environmental stressors, are associated with altered CYP24A1 activity in both systemic and intestinal compartments. Lastly, the gut microbiota—shaped by diet, environment, and disease—contributes additional regulatory input. Microbial metabolites can enhance or suppress vitamin D signaling, depending on microbial composition and host condition. Altogether, these factors converge to create a complex and adaptive network regulating intestinal CYP24A1 expression in support of mineral homeostasis and mucosal health [4,12,17,50,53].

### 4.6. Evidence from Genetic Models of Vitamin D Regulation

Knockout models have provided key insights into the intestinal functions of CYP24A1. Global Cyp24a1 KO mice show excessive accumulation of 1,25(OH)_2_D_3_, altered calcium–phosphate balance, and high early mortality, demonstrating the enzyme’s role in controlling active vitamin D levels. Intestinal-specific KO models reveal that local CYP24A1 governs mucosal responses and expression of calcium transporters, independently of systemic hormone levels. Supporting evidence from VDR and CYP27B1 KO mice confirms the essential role of vitamin D signaling in calcium absorption and epithelial function. These models highlight that CYP24A1 activity is shaped by transcriptional networks, dietary calcium, and the microbial environment. Overall, knockout studies emphasize CYP24A1’s dual role as a systemic and local modulator of vitamin D metabolism in the gut [12,70].

In summary, CYP24A1 expression in the small intestine is governed by a multifaceted regulatory network that integrates nuclear receptor signaling, inflammatory pathways, microbial cues, hormonal inputs, and environmental factors. Together, these mechanisms enable dynamic, tissue-specific modulation of vitamin D catabolism in response to physiological demand and pathological stress. Understanding these regulatory layers is essential for evaluating the enzyme’s role in health and disease and for informing future therapeutic strategies.

## 5. CYP24A1 and Gastrointestinal Disorders

**Figure 4:**
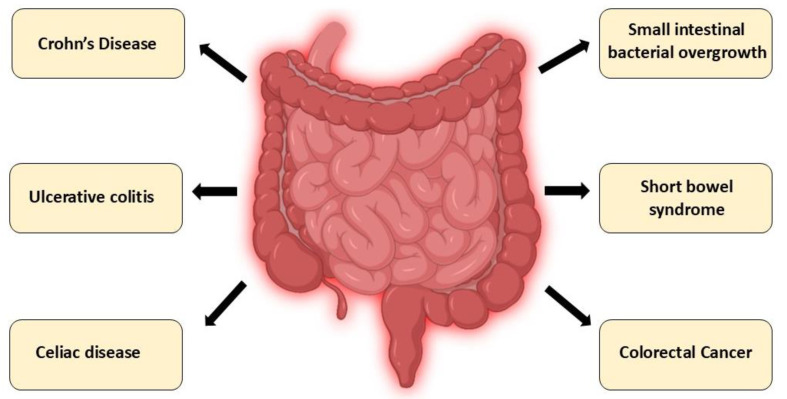
Intestinal disorders associated with CYP24A1 dysregulation: A schematic overview of gastrointestinal disorders in which dysregulation of CYP24A1 expression or activity has been implicated. These include chronic inflammatory conditions (Crohn’s disease, ulcerative colitis), immune-mediated enteropathies (celiac disease), dysbiosis-related syndromes (small intestinal bacterial overgrowth), malabsorptive conditions (short bowel syndrome), and colorectal cancer. Altered CYP24A1 function in these diseases contributes to impaired vitamin D metabolism, disrupted calcium–phosphate balance, compromised epithelial integrity, and dysregulated immune responses.

Altered intestinal expression of CYP24A1 has been implicated in gastrointestinal diseases through its effects on local vitamin D catabolism, epithelial barrier function, and immune signaling. Below, we highlight the role of CYP24A1 in selected intestinal disorders, including IBD, celiac disease, and colorectal cancer (Figure 4, Table 2).

### 5.1. Inflammatory Bowel Diseases

#### 5.1.1. Crohn’s Disease

Crohn’s disease (CD) is a chronic inflammatory condition that can affect any part of the gastrointestinal tract. It is characterized by transmural inflammation, granuloma formation, and complications such as strictures and fistulas. Recent data implicate overexpression of CYP24A1 in the intestinal mucosa as a contributing factor to disease progression. Studies in CD patients have shown CYP24A1 mRNA expression to be elevated 3- to 5-fold in inflamed mucosa compared to controls [71,72]. Pro-inflammatory cytokines, particularly TNF-α and IFN-γ, appear to upregulate CYP24A1, accelerating local catabolism of 1,25(OH)_2_D_3_ and diminishing VDR-mediated transcription of protective genes, including PTPN2. The resulting reduction in PTPN2 promotes claudin-2 expression, increasing paracellular permeability and amplifying mucosal injury. The interaction between inflammation, gut microbiota, and vitamin D metabolism is bidirectional: microbial-derived metabolites and immune cell cytokines further influence CYP24A1 levels, creating a vicious cycle of dysregulation. Vitamin D deficiency is common in CD due to malabsorption and mucosal damage, and correlates with increased disease activity and poor response to supplementation. As such, pharmacological inhibition of CYP24A1 may represent a promising approach to restore local vitamin D activity, enhance epithelial barrier function, and promote mucosal healing [12,71,72,73,74,75,76].

Mechanistic summary: In CD, pro-inflammatory cytokines (TNF-α, IFN-γ) upregulate CYP24A1, accelerating 1,25(OH)_2_D_3_ degradation and reducing VDR-mediated transcription of barrier-protective genes, thereby amplifying mucosal injury through increased paracellular permeability.

#### 5.1.2. Ulcerative Colitis

Ulcerative colitis (UC), confined to the colon and rectum, is marked by continuous superficial inflammation of the mucosal layer. Although the disease is distinct from CD in its pathology, it shares similar disruptions in vitamin D signaling. Elevated levels of IL-6 and IL-1β in the inflamed mucosa may promote aberrant CYP24A1 expression, leading to excessive degradation of 1,25(OH)_2_D_3_ and reduced VDR signaling. While the inflammatory milieu in UC shares similarities with CD—including elevated pro-inflammatory cytokines and VDR suppression—the cytokine profile differs somewhat, with IL-6 and IL-1β being more prominent in UC compared to the TNF-α and IFN-γ dominance in CD. However, the fundamental mechanism of CYP24A1 dysregulation through inflammation-mediated VDR suppression appears largely conserved between the two conditions, though the extent and pattern of dysregulation may vary with disease severity and anatomical distribution. Consequently, the expression of epithelial junctional proteins such as claudin-4 and occluding is diminished, weakening the mucosal barrier and facilitating bacterial translocation. Microbiota dysbiosis further contributes to this dysregulation, as changes in microbial composition alter the production of short-chain fatty acids and other metabolites that can modulate CYP24A1 expression. Moreover, individual variation in CYP24A1 expression may arise from promoter methylation or genetic polymorphisms. Taken together, these mechanisms highlight CYP24A1 as a central player in UC pathophysiology and a potential therapeutic target for enhancing mucosal defense and immune regulation through the vitamin D axis [17,65,73,77,78].

Mechanistic summary: In UC, elevated IL-6 and IL-1β promote aberrant CYP24A1 expression, leading to excessive 1,25(OH)_2_D_3_ degradation, diminished epithelial junction protein expression, and compromised mucosal barrier function.

### 5.2. Celiac Disease and Malabsorption Syndromes

Celiac disease is a chronic, immune-mediated enteropathy triggered by gluten ingestion in genetically predisposed individuals. Characterized by villous atrophy, crypt hyperplasia, and lymphocytic infiltration, it leads to impaired absorption of nutrients, including fat-soluble vitamins such as vitamin D. Notably, even patients adhering to a strict gluten-free diet frequently present with persistent vitamin D deficiency, pointing to mechanisms beyond malabsorption. Inflammatory cytokines and epithelial injury associated with active disease states may induce CYP24A1 expression in enterocytes, increasing the degradation of 1,25-dihydroxyvitamin D_3_ [1,25(OH)_2_D_3_] and weakening its protective effects on mucosal immunity and barrier integrity [9,12,18,53,73].

Vitamin D plays a key immunomodulatory role in regulating T cell responses and dendritic cell function; its depletion—exacerbated by CYP24A1 upregulation—may contribute to persistent inflammation and barrier dysfunction. This dysregulation can also result in systemic consequences, including hypocalcemia, secondary hyperparathyroidism, and metabolic bone disease. In refractory cases, standard supplementation may prove insufficient, highlighting the therapeutic potential of CYP24A1 inhibition to restore local hormone bioavailability, support mucosal recovery, and prevent further complications [79,80,81].

In malabsorptive conditions such as celiac disease, epithelial damage and chronic inflammation reduce vitamin D absorption, triggering compensatory mechanisms aimed at preserving systemic mineral balance. One such response involves the downregulation of CYP24A1 to limit the degradation of 1,25(OH)_2_D_3_ and sustain its biological activity. This adaptive suppression is shaped by interactions with CYP27B1, PTH, FGF23, and inflammatory mediators. However, in the presence of dysbiosis or prolonged inflammation, microbial metabolites and immune signals may unpredictably modulate CYP24A1 expression, potentially destabilizing local vitamin D homeostasis [17,20,26,82].

Mechanistic summary: In celiac disease, inflammatory cytokines and epithelial injury induce CYP24A1 expression, increasing 1,25(OH)_2_D_3_ degradation and weakening mucosal immunity and barrier integrity, even in patients adhering to gluten-free diets.

### 5.3. Small Intestinal Bacterial Overgrowth and Microbiota Dysbiosis

Small intestinal bacterial overgrowth (SIBO) involves excessive bacterial proliferation in the small intestine, often following anatomical disruptions like ileocecal valve resection, enabling retrograde colonization by colonic microbes [83,84,85,86,87,88,89,90]. Gram-negative bacteria predominate, leading to carbohydrate fermentation, gas production, diarrhea, and motility disturbances. Predisposing factors include hypochlorhydria, chronic PPI use, *Helicobacter pylori* infection, and mucosal damage such as villous atrophy [91,92,93]. A hallmark of SIBO is dysbiosis—an imbalance of microbial communities—which impairs nutrient absorption, immune regulation, and barrier integrity. Overgrowth of fermenting bacteria (e.g., *Bacteroides*, *Lachnospira*) and loss of protective species (e.g., *Lactococcus*) fuel mucosal inflammation. Recent studies suggest that dysbiosis alters local vitamin D metabolism. While 1,25-dihydroxyvitamin D_3_ [1,25(OH)_2_D_3_] supports tight junction integrity and immune tolerance, inflammation-induced CYP24A1 expression may enhance its degradation. Conversely, microbial metabolites like butyrate may suppress CYP24A1, indicating a bidirectional relationship between microbiota and vitamin D signaling. These insights position CYP24A1 as a potential mediator between microbial composition, epithelial homeostasis, and immune responses in SIBO. Further studies are needed to clarify its role in dysbiosis-related pathologies [12,38,44,50,89,94].

Mechanistic summary: In SIBO, dysbiosis-driven inflammation likely enhances CYP24A1-mediated degradation of 1,25(OH)_2_D_3_, while loss of beneficial microbial metabolites may further dysregulate local vitamin D signaling, compromising epithelial homeostasis.

### 5.4. Short Bowel Syndrome and Intestinal Adaptation

Short bowel syndrome (SBS) results from extensive resection of the small intestine, typically leaving less than 180–200 cm—due to Crohn’s disease, ischemia, or malignancy. The consequent loss of absorptive area causes malnutrition, dehydration, and electrolyte imbalance. Outcomes depend on residual anatomy, especially retention of the ileocecal valve and colon, which enhance adaptation [95,96,97].

Post-resection, the gut undergoes structural and functional remodeling, including mucosal hypertrophy, villus elongation, and transporter upregulation, driven by hormonal, luminal, and microbial signals. The colon, when present, ferments undigested carbohydrates into short-chain fatty acids, supporting energy balance and mucosal integrity. In contrast, patients with end-jejunostomies often experience more severe metabolic complications [98,99].

Although direct data are limited, dysregulated CYP24A1 may impair vitamin D signaling during adaptation, reducing calcium absorption and epithelial repair. Microbiota shifts and inflammation could further modulate CYP24A1 expression, altering active vitamin D availability. Deciphering how CYP24A1 integrates microbial, inflammatory, and epithelial stress cues may offer novel insights into adaptation mechanisms and therapeutic strategies in SBS [38,39,71,100].

Mechanistic summary: In SBS, dysregulated CYP24A1 expression—modulated by microbiota shifts and inflammation—may impair vitamin D signaling during intestinal adaptation, reducing calcium absorption and compromising epithelial repair capacity.

### 5.5. Colorectal Cancer: The Role of CYP24A1 in Tumorigenesis

CYP24A1, encoding the mitochondrial enzyme 24-hydroxylase, plays a paradoxical role in colorectal cancer (CRC)—acting both as a physiological regulator of vitamin D metabolism and a pathological driver of tumor progression. Under normal conditions, it prevents hypervitaminosis D by degrading excess 1,25-dihydroxyvitamin D_3_ [1,25(OH)_2_D_3_]. In CRC, however, its overexpression impairs vitamin D–mediated tumor-suppressive functions such as cell cycle arrest, differentiation, apoptosis, and inhibition of angiogenesis and metastasis. In colorectal adenomas and carcinomas, CYP24A1 protein expression is elevated 2- to 4-fold compared to normal mucosa, and this correlates with proliferation markers (Ki-67) and reduced patient survival [101,102,103]. Elevated CYP24A1 levels in colorectal adenomas and carcinomas correlate with proliferation markers and poor prognosis, and contribute to resistance to calcitriol-based therapies by promoting hormone inactivation. In vitro studies show that pharmacological inhibition of CYP24A1 restores calcitriol’s antiproliferative effects, underscoring its therapeutic relevance [19,28,29,101,102,103,104,105,106].

Its dysregulation involves both genetic and epigenetic mechanisms, including promoter hypomethylation and regulatory SNPs that may influence CRC risk and treatment response. Inflammatory cytokines and microbial metabolites in the tumor microenvironment further upregulate CYP24A1, creating a feedback loop that blunts vitamin D signaling. Paradoxically, exogenous calcitriol may exacerbate this effect by inducing CYP24A1 expression in deficient tissues. This dual role—protective in physiological contexts yet deleterious in malignancy—positions CYP24A1 as both a biomarker of tumor aggressiveness and a promising therapeutic target in colorectal cancer [28,103,106].

Mechanistic summary: In CRC, CYP24A1 overexpression—driven by genetic, epigenetic, and inflammatory mechanisms—accelerates 1,25(OH)_2_D_3_ degradation, impairing vitamin D-mediated tumor-suppressive functions and contributing to therapeutic resistance.

## 6. Therapeutic Perspectives and Future Directions

Therapeutic modulation of CYP24A1 has emerged as a promising strategy for managing disorders marked by impaired vitamin D signaling and disrupted calcium–phosphate balance. Selective inhibition of intestinal CYP24A1 may enhance local 1,25-dihydroxyvitamin D_3_ [1,25(OH)_2_D_3_] activity and promote calcium absorption without causing systemic hypercalcemia—a major limitation of conventional supplementation [10,17,66]. This approach holds particular relevance in conditions such as chronic kidney disease, osteoporosis, nephrolithiasis, and inflammatory bowel disease, where excessive degradation of active vitamin D contributes to disease progression. Pharmacological inhibitors of CYP24A1—including azole antifungals, imidazole derivatives (e.g., VID-400), and newer selective compounds (e.g., CTA-018)—aim to preserve circulating and tissue-level vitamin D metabolites. Among these, ketoconazole and other azole antifungals have shown CYP24A1 inhibitory activity but lack specificity and carry significant off-target effects. VID-400, an imidazole derivative, has advanced to preclinical testing and demonstrates improved selectivity, achieving approximately 60% inhibition of CYP24A1 activity in animal models. CTA-018, a newer selective compound, exhibits >80% selectivity for CYP24A1 over other cytochrome P450 enzymes and has shown promise in early-phase studies. However, challenges remain including limited oral bioavailability, hepatic first-pass metabolism, and the activation of compensatory pathways such as glucuronidation that may reduce therapeutic efficacy [107,108,109,110,111,112,113,114,115,116]. To date, no CYP24A1 inhibitor has received regulatory approval for clinical use, though several candidates are in Phase I/II development for chronic kidney disease and osteoporosis. However, challenges remain, including off-target effects, limited specificity, and compensatory metabolic pathways such as glucuronidation that may reduce therapeutic efficacy [107,108,109,110,111,112,113,114,115,116]. In parallel, dietary and microbiome-based strategies—such as prebiotic and probiotic interventions—seek to indirectly modulate CYP24A1 by altering local inflammatory signals, microbial composition, and epithelial responsiveness to vitamin D. Regarding microbiome-based strategies, while prebiotics and probiotics can modulate inflammatory signals and SCFA production, the direct quantitative impact on intestinal CYP24A1 expression remains incompletely characterized and likely varies with baseline microbiome composition and host genetics [5,12,17,18,44,55,58,59,94,117,118,119,120,121,122,123]. Emerging genetic tools offer additional precision. Gene therapy and siRNA-based approaches enable tissue-specific downregulation of CYP24A1 expression, providing long-term modulation of vitamin D metabolism in the intestinal mucosa [5,12,17,18,44,55,58,59,94,117,118,119,120,121,122,123]. Artificial intelligence (AI) further enhances personalization by integrating clinical, genomic, and environmental data to model individual vitamin D requirements and optimize dosing. Specifically, AI-driven algorithms could integrate patient-specific data—such as CYP24A1 and VDR polymorphisms, microbiome composition profiles, serum inflammatory markers, and baseline vitamin D metabolite levels—to create predictive models that identify individuals most likely to benefit from CYP24A1 inhibition or personalized vitamin D dosing strategies. When paired with sensory analysis, AI can also inform the design of palatable delivery systems, improving adherence and clinical outcomes [124,125,126,127,128,129,130,131].

**Figure 5:**
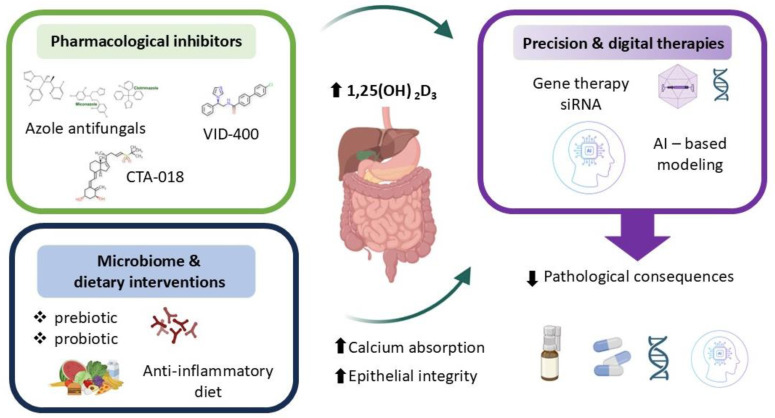
Therapeutic perspectives.

Together, these innovative strategies reflect a shift toward personalized, mechanism-based interventions. By aligning molecular targeting of CYP24A1 with patient-specific factors, future therapies may more effectively restore intestinal vitamin D activity, support epithelial integrity, and improve systemic mineral homeostasis (Figure 5).

## 7. Unresolved Questions and Experimental Approaches

### 7.1. Controversies and Limitations

Despite growing evidence supporting CYP24A1 as a therapeutic target, several controversies and limitations warrant acknowledgment. First, the role of CYP24A1 in cancer appears paradoxical: while physiologically protective against hypervitaminosis D, its overexpression in malignancies such as colorectal cancer drives tumor progression by inactivating the antiproliferative effects of 1,25(OH)_2_D_3_. Conversely, in some contexts, CYP24A1 may protect against vitamin D toxicity during high-dose supplementation or calcitriol therapy, raising questions about when inhibition is appropriate.

Conflicting results exist regarding inflammation-induced regulation of CYP24A1. Some studies report upregulation in response to TNF-α and IL-6, while others describe suppression during acute inflammatory responses, possibly reflecting differences in timing, cell type, or disease stage. These inconsistencies complicate the development of unified therapeutic strategies.

Systemic blockade of CYP24A1 carries risks, including hypercalcemia, nephrocalcinosis, and soft tissue calcification. Tissue-specific inhibition remains technically challenging, and the long-term safety profile of chronic CYP24A1 suppression is unknown. Additionally, compensatory upregulation of alternative catabolic pathways may limit therapeutic efficacy.

Most mechanistic data derive from animal models, and translational applicability to human intestinal physiology remains uncertain. Large-scale clinical trials are needed to validate CYP24A1-targeted interventions and define patient populations most likely to benefit.

### 7.2. Key Knowledge Gaps and Future Research Directions

Despite growing interest in CYP24A1’s role in intestinal vitamin D metabolism, key gaps remain. The full spectrum of factors regulating its expression in enterocytes—beyond calcitriol–VDR signaling—requires clarification, especially the roles of microbial metabolites and inflammatory signals. Although microbiota shifts have been linked to altered vitamin D pathways, direct causal links to CYP24A1 are not well established. Controlled systems, including gnotobiotic mice and human intestinal organoids, are needed to address this. The contribution of intestinal CYP24A1 to systemic calcium and phosphate homeostasis is also unclear, compensatory mechanisms seen in VDR-deficient models suggest possible redundancy that should be tested using intestinal CYP24A1 knockout or overexpression strategies combined with tracer-based flux studies.

Therapeutically, inhibiting intestinal CYP24A1 may enhance local calcitriol effects without systemic toxicity, but current inhibitors lack full specificity. Broader physiological effects, such as on immune or skin phenotypes, highlight the need for comprehensive phenotyping and Mendelian randomization approaches. Emerging tools—including CRISPR/Cas9 gene editing, single-cell transcriptomics, metabolomics, and intestinal organoids—offer promising platforms to explore CYP24A1 regulation in specific epithelial populations and disease contexts. Finally, further progress will depend on developing selective inhibitors, refined in vivo models, and integrative multi-omics studies supported by cross-disciplinary collaboration.

## 8. Conclusions

CYP24A1, traditionally associated with systemic vitamin D metabolism, is increasingly recognized as a key regulator of local vitamin D signaling in the small intestine. Its expression in enterocytes responds dynamically to hormonal, microbial, inflammatory, and epigenetic cues, enabling the intestine to fine-tune calcium and phosphate absorption in response to changing physiological demands and pathological insults. Disruption of this regulatory balance has been implicated in several gastrointestinal disorders, including inflammatory bowel disease, celiac disease, colorectal cancer, and malabsorption syndromes, where altered CYP24A1 activity may compromise epithelial barrier function, immune modulation, and nutrient transport.

Mounting evidence also points to the therapeutic relevance of modulating intestinal CYP24A1. Targeting this enzyme, whether through selective inhibitors, gene silencing techniques, or microbiota-directed strategies, holds promise for enhancing local vitamin D bioactivity without provoking systemic side effects. However, important knowledge gaps remain, particularly regarding the systemic consequences of intestinal CYP24A1 modulation, its cell-type specificity within the gut epithelium, and the long-term safety and efficacy of therapeutic interventions.

Advancing this field will require interdisciplinary approaches that combine high-resolution experimental models, multi-omics analyses, and precision-targeted tools. A deeper understanding of CYP24A1’s dual role as both a local regulator of vitamin D catabolism and a potential driver of intestinal disease may offer new opportunities for translational research and contribute to the development of personalized therapies in gastrointestinal and mineral metabolism disorders.

## Figures and Tables

**Table 1:** Comparison of CYP24A1 Functions in Kidney versus Small Intestine.

Feature	Renal CYP24A1	Intestinal CYP24A1
Primary function	Systemic vitamin D clearance; maintains circulating 1,25(OH)_2_D_3_ levels	Local regulation of mucosal vitamin D activity; modulates calcium absorption
Basal expression	High constitutive expression	Low basal expression, highly inducible
Inducibility	Moderate (up to 10-fold)	Extreme (up to 370,000-fold in duodenum)
Main regulators	PTH (−), FGF23 (+), 1,25(OH)_2_D_3_ (+)	1,25(OH)_2_D_3_ (+), dietary calcium, inflammatory cytokines, microbiota
Regulatory complexity	Primarily endocrine	Endocrine + paracrine + luminal + microbial
Knockout phenotype	Systemic hypervitaminosis D, hypercalcemia	Altered mucosal vitamin D signaling, disrupted local calcium transport
Therapeutic target	CKD, hypercalcemia, FGF23 excess	IBD, celiac disease, malabsorption, colorectal cancer
Clinical implications	Systemic mineral balance	Epithelial integrity, immune modulation, nutrient absorption

**Table 2:** Summary of Intestinal CYP24A1 Dysregulation in Gastrointestinal Disorders.

Disorder	CYP24A1 Status	Key Mechanisms	Clinical Consequences
**Crohn’s Disease**	↑↑ (3–5-fold)	TNF-α, IFN-γ upregulation; VDR suppression	Increased mucosal permeability, barrier dysfunction, reduced PTPN2 expression
**Ulcerative Colitis**	↑↑	IL-6, IL-1β elevation; VDR signaling impairment	Weakened tight junctions, bacterial translocation, inflammation
**Celiac Disease**	↑	Inflammatory cytokines; epithelial injury	Persistent vitamin D deficiency despite GFD, impaired mucosal immunity
**SIBO/Dysbiosis**	↑ (presumed)	Dysbiosis, LPS, loss of SCFAs	Compromised epithelial integrity, immune dysregulation
**Short Bowel Syndrome**	Variable (↑/↓)	Microbiota shifts, inflammation, adaptive stress	Impaired calcium absorption, reduced epithelial adaptation
**Colorectal Cancer**	↑↑↑ (2–4-fold)	Promoter hypomethylation, SNPs, tumor microenvironment	Tumor proliferation, resistance to calcitriol therapy, poor prognosis

**Note:** ↑ = elevated; ↑↑ = markedly elevated; ↑↑↑ = highly elevated; ↓ = decreased; GFD = gluten-free diet; SIBO = small intestinal bacterial overgrowth; SCFAs = short-chain fatty acids; SNPs = single-nucleotide polymorphisms.

## Data Availability

Not applicable.
